# Strontium, oxygen, and carbon isotope variation in modern human dental enamel

**DOI:** 10.1002/ajpa.24059

**Published:** 2020-04-25

**Authors:** Esther Plomp, Isabella C. C. von Holstein, Lisette M. Kootker, Suzanne J. A. Verdegaal‐Warmerdam, Tim Forouzanfar, Gareth R. Davies

**Affiliations:** ^1^ Vrije Universiteit Amsterdam, Department of Earth Sciences Amsterdam Netherlands; ^2^ Delft University of Technology, Faculty of Applied Sciences Delft Netherlands; ^3^ Co van Ledden Hulsebosch Center (CLHC) Amsterdam Netherlands; ^4^ Amsterdam University Medical Centers (Amsterdam UMC), location VUmc, Department of Oral and Maxillofacial surgery Amsterdam Netherlands; ^5^ Academic Centre for Dentistry Amsterdam (ACTA) Amsterdam Netherlands

**Keywords:** human, isotopes, strontium, oxygen, carbon

## Abstract

**Objectives:**

Isotopic analyses using human dental enamel provide information on the mobility and diet of individuals in forensic and archeological studies. Thus far, no study has systematically examined intraindividual coupled strontium (Sr), oxygen (O), and carbon (C) isotope variation in human enamel or the effect that caries have on the isotopic integrity of the enamel. The inadequate quantification of isotopic variation affects interpretations and may constrain sample selection of elements affected by caries. This study aims to quantify the intraindividual isotopic variation and provides recommendations for enamel sampling methods.

**Material and Methods:**

This study presents the first systematic results on intraindividual variation in Sr–O–C isotope composition and Sr concentration in modern human dental enamel of third molars (affected and unaffected by caries). A multiloci sampling approach (*n* = 6–20) was used to analyze surface and inner enamel, employing thermal ionization mass spectrometry (TIMS) and isotope ratio mass spectrometry (IRMS). Third molars were analyzed from 47 individuals from the Netherlands, Iceland, the United States, the Caribbean, Colombia, Somalia, and South Africa.

**Results:**

Intradental isotopic variation in modern Dutch dental elements was recorded for Sr, O, and C and exceeded the variation introduced by the analytical error. Single loci and bulk sampling approaches of third molars established that a single analysis is only representative of the bulk Sr isotope composition in 60% of the elements analyzed. Dental elements affected by caries showed twice the variation seen in unaffected dental elements. Caries did not consistently incorporate the isotopic composition of the geographical environment in which they developed.

**Discussion:**

The isotopic variability recorded in unaffected inner enamel indicates that variations greater than 0.000200 for ^87^Sr/^86^Sr and larger than 2‰ for δ^18^O and δ^13^C are required to demonstrate changes in modern Dutch human diet or geographic location.

## 1 INTRODUCTION

Human dental enamel contains information regarding the geographical origin and dietary patterns of an individual. Dental enamel records the isotopic signatures of the diet consumed during enamel mineralization (Bentley, 2006; Lee‐Thorp, 2008; Montgomery, 2002), which for the permanent dentition takes place between birth and circa 16 years of age (AlQahtani, Hector, & Liversidge, 2010; Nanci, 2012; Piesco & Avery, 2002). The isotopic signature of the enamel, representative of the diet and geographical location of the individual during childhood, is preserved because enamel does not remodel after mineralization. Furthermore, enamel is markedly resistant to diagenetic alteration as it is a highly mineralized tissue, with low organic content and porosity (Hoppe, Koch, & Furutani, 2003; Kohn, Schoeninger, & Barker, 1999; Lee‐Thorp & Sponheimer, 2003; Nanci, 2012; Piesco & Simmelink, 2002). Strontium (Sr), oxygen (O), and carbon (C) isotope analyses are established techniques to infer information about human provenance (^87^Sr/^86^Sr (e.g., Bentley, 2006), δ^18^O (e.g., Blumenthal et al., 2014; Bowen, 2010; Lightfoot & O'Connell, 2016; Pellegrini, Pouncett, Jay, Pearson, & Richards, 2016); and diet (δ^13^C, for example, Chesson et al., 2018; France & Owsley, 2015; Lee‐Thorp, 2008). These isotopic analyses have been successfully applied to both modern (e.g., Font, Van Der Peijl, Van Leuwen, Van Wetten, & Davies, 2015; Vautour, Poirier, & Widory, 2015) and archeological individuals (e.g., Bataille et al., 2018; Kootker, Mbeki, Morris, Kars, & Davies, 2016; Laffoon et al., 2017; Panagiotopoulou et al., 2018; Snoeck et al., 2018).

The isotopic variation in human tissues is widely used as an indicator for diet and migration. However, the relationship between intraindividual and intrapopulation variation has not been adequately quantified in humans despite considerable geochemical variability in enamel demonstrated by previous studies (Hare, Austin, Doble, & Arora, 2011; Smith et al., 2018; Willmes et al., 2016; Wright, 2013). Both types of variation will be related to the variability of isotopic inputs over the time of enamel formation. Intraindividual variation may, however, also be related to (a) sampling location, (b) sampling method, and (c) enamel damage, specifically caries.

The analysis of strontium using thermal ionization mass spectrometry (TIMS) and oxygen and carbon isotopes using isotope ratio mass spectrometry (IRMS) is now widespread in archeological and forensic research due to the precision and accuracy that these analysis techniques provide (Slovak & Paytan, 2011). Multiloci sampling methods (e.g., Craig‐Atkins, Towers, & Beaumont, 2018) are generally not applied on human enamel using TIMS and IRMS, however, because the final stage of enamel formation, mineralization, has been suggested to overwrite any record of the isotopic composition of the initial incremental enamel deposition (Fincham, Moradian‐Oldak, & Simmer, 1999; Montgomery, Evans, & Wildman, 2006; Montgomery, 2002; Montgomery, Evans, & Cooper, 2007; Müller et al., 2019; Trayler & Kohn, 2017; Zazzo, Balasse, & Patterson, 2005). This re‐equilibration of the isotopic composition during the mineralization phase means that the isotopic composition of the sample taken along the incremental enamel layers would be representative of the isotopic composition during mineralization rather than incremental enamel deposition (Montgomery & Evans, 2006; Trayler & Kohn, 2017). Moreover, sequential sampling is hampered by a poor understanding of enamel formation and mineralization. The effects of spatial and temporal controls as well as physiological factors (e.g., health, sex, diet, and physical activity) are unknown (Balasse, 2003; 2002; Blumenthal et al., 2014; Fincham et al., 1999; Reade, Stevens, Barker, & O'Connell, 2015; Simmer & Fincham, 1995; Trayler & Kohn, 2017). In addition, the effect that these physiological factors have on enamel formation differs in various populations (Tompkins, 1996). Isotopic values incorporated in the enamel are also influenced by geographical controls, especially O and Sr, which are controlled by the local precipitation (O, Lightfoot & O'Connell, 2016) and geology (Sr, Bentley, 2006). An example of temporal control is the introduction of the modern supermarket diet in the 1970s. The availability of a greater variety of products grown in different geological settings is expected to increase the isotopic variation seen in modern human dental enamel compared to archeological dental enamel (Chesson, Ehleringer, & Cerling, 2011; Valenzuela, Chesson, Bowen, Cerling, & Ehleringer, 2012; Vautour et al., 2015).

Currently, no formal guidelines have been established for enamel sampling methods used in isotopic studies of human dental elements. The lack of a formal sampling approach may affect the intraindividual variation seen in individuals sampled as well as decrease the comparability between isotopic analyses of studies that use different sample loci. Presently, enamel sampling generally involves a single sample location of a dental element, collected across a tooth's inner enamel, indiscriminate of enamel growth phases (Montgomery & Evans, 2006; Slovak & Paytan, 2011). This sampling approach disregards the potential influence of intraindividual isotopic variation within a single dental element, that is, intradental variation. It is therefore unknown if a single sample location is representative of the total enamel Sr–O–C isotope composition of the dental element, referred to in this study as the bulk isotopic composition. Enamel is generally sampled using a handheld dental drill with tungsten or diamond burrs or saws (e.g., Balasse, 2003; Slovak & Paytan, 2011; Trayler & Kohn, 2017). Some studies suggest that diagenetic Sr contaminates the surface enamel (~0.1 mm) after mineralization due to diffusion of Sr in the saliva from the diet and water in the mouth (Dufour et al., 2007; Horn & Müller‐Sohnius, 1999) or because of interaction with the burial environment (Kohn et al., 1999; Schoeninger, Hallin, Reeser, Valley, & Fournelle, 2003). As a result, tooth surfaces are usually mechanically cleaned by removing the outer surface layer prior to sampling (Balasse, 2002; Kootker, van Lanen, Kars, & Davies, 2016; Reade et al., 2015; Slovak & Paytan, 2011). Although detailed sampling information is rarely provided in scientific literature, researchers seem to prefer to avoid sampling surface enamel in contact with other dental elements or areas that are affected by caries or other defects (Kootker, Mbeki, et al., 2016; Montgomery, 2002). Therefore, the potential influence of carious processes, such as demineralization and remineralization (see references in Li, Wang, Joiner, and Chang (2014) and Cochrane, Saranathan, Cai, Cross, and Reynolds (2008)), on Sr–O–C isotope ratios remains unknown.

A better understanding of the intradental isotopic variation of nonmigratory individuals is therefore required to provide a baseline of intraindividual isotopic variation. This will improve the accuracy of the interpretation of mobility and dietary patterns in both archeological and forensic contexts. Therefore, this study evaluated intraindividual isotope variation of Sr–O–C isotope composition, as well as Sr concentration, within modern human dental enamel of third molars (affected and unaffected by caries) from individuals known to have lived in one location during enamel formation and mineralization.

This study aims to determine if:

1. The variation of Sr–O–C isotope composition in modern human dental enamel is in the same order of magnitude between various sample locations within the same dental element (intradental), as well as within other dental elements (interdental) of the same individual. By quantifying the intradental variation, it could be examined if there is any spatial control on the Sr–O–C isotope composition within a dental element.
2. An individual's life history (year and region of birth) has an effect on the Sr–O–C isotope variation in modern human enamel.
3. The Sr–O–C isotope values of single sample locations from the cusp match the average isotopic variation obtained from bulk sample analysis of the same dental element.
4. The presence of caries affects the variation seen in Sr–O–C isotope composition of modern human dental elements.

Finally, these data are used to recommend a sampling protocol and to establish what Sr–O–C isotope variation is required to establish dietary change or mobility in modern Dutch humans.

## 2 MATERIALS AND METHODS

### 2.1 Sample selection

**FIGURE 1:**
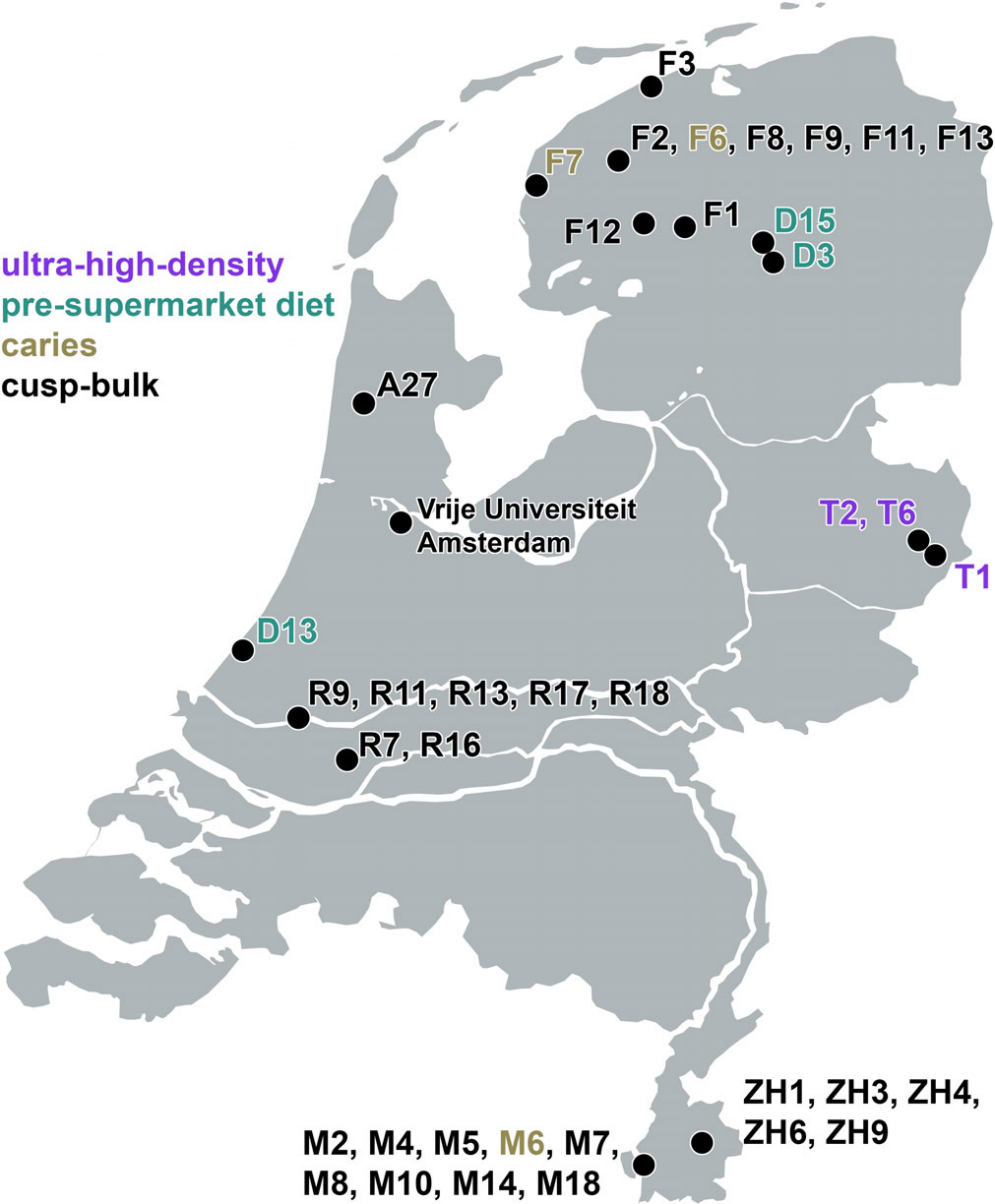
A map of the Netherlands with localities where the individuals (*n* = 38) lived during formation of their third molars. F3 = Holwerd; F2, F6, F8, F9, F11, F13 = Leeuwarden; F7 = Wijnaldum; F12 = Oldeboorn; F1 = Lippenhuizen; D15 = Veenhuizen; D3 = Smilde; A27 = Alkmaar; 9 = Amsterdam; T2, T6 = Hengelo; T1 = Enschede; D13 = Den Haag; R9, R11, R13, R17, R18 = Rotterdam; R7 = Dordrecht; ZH1, ZH3, ZH4, ZH6, ZH9 = Heerlen; M2, M4, M5, M6, M7, M8, M10, M14, and M18 = Maastricht

Extracted third molars were donated to the Vrije Universiteit Amsterdam by patients of dental clinics and medical centers in the Netherlands to be used for isotopic analyses. Background information was obtained through anonymous questionnaires, providing information on an individual's geographical location at the time of enamel formation (Figure 1), as well as diet, health, smoking, and exercise habits. The isotopic analyses of the teeth were approved by the Medical Ethics Review Committee of the VU University Medical Center.

The enamel of third molars is formed between the age of 8 and 16 years (AlQahtani et al., 2010). Teeth were selected based on the mobility profiles of 38 Dutch individuals (residential stability during enamel formation and mineralization) and the presence of caries. In addition, third molars from nine individuals from the Dutch Antilles (*n* = 3, Curaçao, Bonaire, Aruba), the Dominical Republic (*n* = 1), Colombia (*n* = 1), the United States (*n* = 1), Iceland (*n* = 1), Somalia (*n* = 1), and South Africa (*n* = 1) were sampled to compare their isotopic variation with the Dutch isotopic variation.

The following groups were examined:

1. Three individuals born in the same decade in the late 20th century (years of birth 1989, 1995, and 1991, respectively) raised in cities in close proximity to each other in the Netherlands (T1, T2, T6), representative of inputs from a globalized supermarket diet that emerged in the 1970s. For each of these three individuals, one third molar was sampled using an ultrahigh‐density approach (*n* = 20 samples per element), with other third molars sampled using a high‐density approach (*n* = 6 samples per element).
2. Three individuals born in the mid‐20th century (years of birth 1949, 1964, and 1942) from the Netherlands (D3, D13, D15), representative of the pre‐supermarket diet. These individuals were sampled using the high‐density approach (*n* = 6).
3. Single cusp location and bulk sampling approaches were compared for Sr isotope analysis of 35 individuals. The results from the bulk sampling approach were previously reported (Plomp et al., 2019). The results from the Dutch individuals (n = 29) were compared with non‐Dutch individuals (*n* = 6). These individuals were born in the years 1972–1999 and are thus representative of increasing inputs from a globalized supermarket diet.
4. Six individuals whose teeth developed caries. The unaffected enamel of these individuals was sampled using the high‐density approach (*n* = 6), with caries being sampled from the surface toward the inner enamel (*n* = 3–4).

### 2.2 Sample preparation

The enamel was sampled, chemically processed, and analyzed at the Faculty of Science, Vrije Universiteit Amsterdam. Sample preparation and procedures are described in detail in Plomp et al. (2017) and Plomp, Smeets, & Davies (2020). The enamel was sampled perpendicular to the enamel dentine junction (EDJ) using a dental microdrill fitted with an acid cleaned diamond‐tipped rotary burr and blade (Minilor Perceuse). Enamel at the EDJ was not sampled as the thin enamel layer at the EDJ is difficult to isolate (Reade et al., 2015). Occlusal fissures were avoided as they (a) are difficult to mechanically clean and sample and (b) have been reported to be less mineralized (He, Huang, Jing, & Hao, 2010; Montgomery, 2002), making them potentially more prone to diagenesis.

**FIGURE 2:**
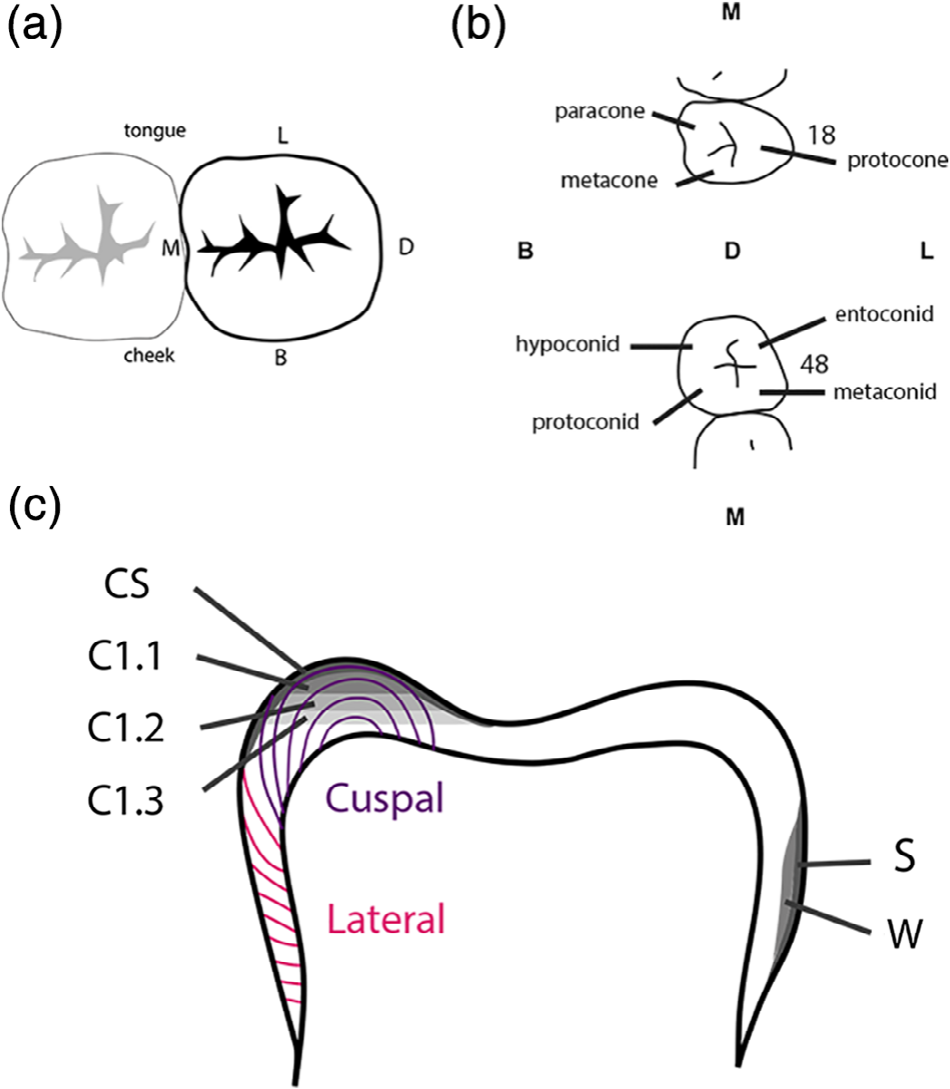
Sample locations in third molars. (a) Occlusal view (from above) indicating L = lingual (toward the tongue), M = mesial (toward the midline point/incisors of the dental arch), D = distal (opposite of mesial, toward the back of the dental arch), B = buccal (toward the cheek). (b) Cusp names (see also Table 1) for maxillary (above) and mandibular (below) third molars. (c) Profile view indicating CS = cusp surface, C1.1 = cusp layer 1, C1.2 = cusp layer 2, C1.3 = cusp layer 3, S = wall surface, and W = wall. Incremental enamel formation is indicated in purple (cuspal) and pink (lateral) lines (after Dean, 2000)

To indicate the sample locations on each molar, a coding system was developed (Table 1 and Figure 2). A distinction was made between the occlusal surface (cusp, or cuspal enamel, Dean, 2000) and the sides of the dental element (wall, or lateral enamel, Dean, 2000) (Figure 2c), where lateral enamel is secreted in a lateral direction and does not contribute to increase the tooth height (Dean, 2000). The tooth wall was sampled on the wall surface (S) and the inner enamel wall (W). Similarly, the tooth cusp was sampled on the surface (CS) and from the inner enamel cusp (C). Sampling at increasing depth for the cusps is indicated by suffix 0.1 to 0.3 (in sample location numbers), representative of 0.2–0.5 mm. To indicate the difference between the four walls, directional terms were used: lingual, buccal, medial, and distal (see Table 1 and Figure 2a). The lingual tooth wall (WL) is next to the tongue, the buccal tooth wall (WB) is opposite the lingual tooth wall, toward the cheek. The mesial tooth wall (WM) is in contact with the second molar toward the midpoint of the dental arch. The distal tooth wall (WD) is opposite the mesial tooth wall toward the back of the dental arch. To indicate the difference between the three to four cusps on the third molar, cusps were classified based on the largest cusp (protocone/protoconid, with names ending in ‐cone indicative for the upper dentition and names ending with ‐id for the lower dentition, see Table 1, Figure 2b). As indicated by the incremental enamel layers in Figure 2c, the samples taken from the surface cusps (CS1, CS2, CS3, CS4) derive from the same incremental enamel layers. The (surface) wall samples (SL, SB, SM, SD and WL, WB, WM, WD) as well as the inner enamel cusps samples (C1.1, C2.1, C3, C4) cross multiple incremental enamel layers and do not necessarily represent the same or distinct time periods.

**TABLE 1:** Sample locations for the third molars used in this study

Location	Direction			
Wall	Lingual (L)	Buccal (B)	Mesial (M)	Distal (D)
Wall surface (S)	SL	SB	SM	SD
Wall (W)	WL	WB	WM	WD
Cusp	Protocone/id (1)	Paracone/metaconid (2)	Metacone/entoconid (3)	Hypoconid (4)
Cusp	Mesio‐lingual/buccal	Mesio‐buccal/lingual	Disto‐buccal/lingual	Disto‐buccal
Cusp surface (CS)	CS1	CS2	CS3	CS4
Cusp (C)	C1.1, C1.2, C1.3	C2.1, C2.2, C2.3	C3	C4

Molars with caries were described in a similar way, with carious enamel samples labeled with a G and followed by a number that indicates the increase in depth of sampling toward (and including) unaffected enamel. Some samples had multiple caries that were sampled (e.g., G1.1–G1.3, G2.1–G2.3). The locations of the caries sampled are indicated in Table 2.

**TABLE 2:** Isotopic results of individuals analyzed in this study (*n* = 12)

Sample						Strontium		Oxygen and Carbon	
Individual	City	Birth year	ID	FDI	Sample location	^87^Sr/^86^Sr	ppm	δ^18^O (‰ VPDB)	δ^13^C (‰ VPDB)
Twente 1	Enschede	1989	T1.1	38	SL	0.709785 ± 11	52.0	−6.4	−13.6
					SB	0.709785 ± 9	52.7	−6.5	−13.5
					SM	0.709732 ± 15	49.7	−6.6	−14.1
					SD	0.709785 ± 8	49.2	−6.3	−13.4
					WL	0.709776 ± 9	55.2	−6.5	−14.1
					WB	0.709787 ± 8	56.1	−6.8	−14.1
					WM	0.709871 ± 8	58.0	−6.9	−14.8
					WD	0.709762 ± 9	54.0	−6.1	−14.4
					CS1	0.709848 ± 9	46.6	−5.5	−13.7
					CS2	0.709826 ± 12	47.0	−5.9	−12.9
					CS3	0.709761 ± 11	48.9	−5.8	−12.9
					CS4	0.709772 ± 9	42.3	−6.3	−13.7
					C1.1	0.709884 ± 9	55.8	−6.3	−13.9
					C1.2	0.709877 ± 8	51.5		
					C1.3	0.709879 ± 9	53.6		
					C2.1	0.709890 ± 11	44.6	−6.3	−14.5
					C2.2	0.709890 ± 8	49.2		
					C2.3	0.709885 ± 7	53.7		
					C3	0.709822 ± 9	78.8	−6.2	−13.4
					C4	0.709851 ± 10	52.7	−6.1	−14.4
			T1.2	18	WL	0.709802 ± 9	55.9	−5.8	−13.9
					WB	0.709852 ± 9	50.1	−5.8	−14.0
					WM	0.709883 ± 10	48.5	−6.4	−14.4
					WD	0.709860 ± 9	53.3	−6.7	−14.3
					C1.1	0.709884 ± 8	49.3	−6.0	−14.5
					C3	0.709900 ± 9	52.5	−5.9	−14.6
			T1.3	28	SL			−5.0	−12.8
					SB			−5.7	−12.8
					SM			−5.6	−12.9
					SD			−5.6	−13.1
					WL	0.709891 ± 9	57.2	−6.2	−14.1
					WB	0.709897 ± 8	59.6	−6.2	−14.4
					WM	0.709908 ± 10	54.1	−6.0	−14.5
					WD	0.709864 ± 11	60.3	−5.9	−14.4
					CS1			−5.6	−13.8
					CS2			−5.5	−12.8
					CS3			−5.9	−13.7
					C1.1	0.709872 ± 9	48.3	−6.2	−14.6
					C2.1			−5.8	−14.8
					C3	0.709877 ± 9	33.9	−5.9	−14.9
Twente 2	Hengelo	1995	T2.1	48	SL	0.709294 ± 10	37.6	−7.1	−13.4
					SB	0.709304 ± 8	36.8	−7.2	−13.3
					SM	0.709313 ± 10	39.1	−6.4	−13.5
					SD	0.709303 ± 9	37.2	−6.4	−13.9
					WL	0.709266 ± 8	39.1	−7.2	−14.1
					WB	0.709279 ± 10	37.5	−7.2	−13.6
					WM	0.709295 ± 8	38.3	−6.6	−14.3
					WD	0.709312 ± 10	38.6	−6.5	−14.3
					CS1	0.709294 ± 9	31.1	−5.2	−12.8
					CS2	0.709329 ± 8	37.3	−6.0	−13.7
					CS3	0.709338 ± 10	37.9	−6.4	−13.9
					CS4	0.709357 ± 10	36.5	−6.2	−14.1
					C1.1	0.709311 ± 10	36.5	−6.2	−14.4
					C1.2	0.709276 ± 8	38.1		
					C1.3	0.709299 ± 10	37.5		
					C2.1	0.709302 ± 12	37.8	−6.3	−14.5
					C2.2	0.709293 ± 10	43.8		
					C2.3	0.709282 ± 11	40.1		
					C3	0.709321 ± 7	38.0	−6.4	−14.6
					C4	0.709322 ± 10	36.7	−6.6	−14.2
			T2.2	18	WL	0.709316 ± 10	39.3	−7.0	−14.2
					WB	0.709281 ± 10	42.7	−6.5	−14.3
					WM	0.709311 ± 9	42.1	−6.6	−14.4
					WD	0.709323 ± 7	39.8	−6.5	−14.4
					C1.1	0.709240 ± 9	38.7	−6.1	−14.7
					C2.1			−5.9	−14.2
					C3	0.709287 ± 10	39.2	−6.3	−14.5
Twente 6	Hengelo	1991	T6.1	38	SL	0.709834 ± 9	59.3	−7.4	−14.0
					SB	0.709870 ± 10	69.4	−7.1	−14.2
					SM	0.709843 ± 9	60.8	−7.2	−13.3
					SD	0.709799 ± 9	55.8	−7.0	−14.0
					WL	0.709951 ± 8	74.8	−7.3	−14.5
					WB	0.709830 ± 8	62.6	−7.2	−14.2
					WM	0.709930 ± 9	74.5	−7.1	−14.1
					WD	0.709991 ± 9	61.4	−7.6	−14.7
					CS1	0.709960 ± 13	61.1	−5.7	−12.6
					CS2	0.709988 ± 10	68.9	−6.3	−13.0
					CS3	0.709857 ± 11	59.7	−6.5	−13.3
					CS4	0.709892 ± 10	65.3	−6.5	−13.0
					C1.1	0.709961 ± 10	64.6	−6.5	−14.4
					C1.2	0.709890 ± 10	76.0		
					C1.3	0.709940 ± 10	77.5		
					C2.1	0.709946 ± 9	66.6	−7.2	−14.4
					C2.2	0.709955 ± 9	66.1		
					C2.3	0.709957 ± 10	69.0		
					C3	0.709966 ± 8	68.5	−7.0	−14.3
					C4	0.709909 ± 9	56.5	−6.7	−14.3
			T6.2	28	WL	0.709835 ± 8	84.8	−6.8	−14.1
					WB	0.709817 ± 9	78.5	−6.3	−13.8
					WM	0.709823 ± 9	85.6	−6.5	−14.0
					WD	0.709808 ± 8	78.2	−6.9	−14.1
					C1.1	0.709799 ± 8	88.4	−6.6	−14.4
					C2.1			−6.0	−14.0
					C3	0.709830 ± 9	79.3	−6.0	−13.7
Drenthe 3	Smilde	1949	D3	48	WL	0.709575 ± 10	96.6	−6.1	−12.6
					WB	0.709558 ± 11	93.8	−6.1	−12.7
					WM	0.709552 ± 10	93.7	−6.2	−12.8
					WD	0.709600 ± 9	103.3	−6.9	−12.6
					C1.1	0.709584 ± 10	87.3	−6.2	−12.9
					C2.1	0.709539 ± 10	91.6	−5.5	−12.8
South Holland 13	Den Haag	1942	D13	48	WL	0.709202 ± 9	96.2	−6.1	−13.5
					WB	0.709210 ± 7	95.9	−5.9	−14.0
					WM	0.709202 ± 9	100.6	−6.0	−13.8
					WD	0.709223 ± 7	97.9	−6.3	−13.9
					C1.1	0.709216 ± 9	94.6	−6.3	−13.9
					C2.1	0.709208 ± 8		−6.0	−14.0
Drenthe 15	Veenhuizen	1964	D15	38	WL	0.709412 ± 7	100.6	−6.4	−13.5
					WB	0.709392 ± 8	64.3	−6.1	−13.7
					WM	0.709383 ± 9	60.8	−6.0	−14.0
					WD	0.709392 ± 10	65.2	−6.6	−13.7
					C1.1	0.709395 ± 10	56.8	−5.9	−13.8
					C2.1	0.709405 ± 10	57.8	−6.0	−13.8
Friesland 6	Leeuwarden	1986	F6	28	WM	0.709583 ± 9	50.3	−6.2	−13.7
					C1.1	0.709535 ± 9	49.5	−5.8	−13.8
					G1.1 WL	0.709586 ± 10	54.3	−5.4	−13.8
					G1.2 WL	0.709605 ± 10	42.3		
					G1.3 WL	0.709587 ± 10	57.3		
					G1.4 WL	0.709571 ± 11	49.0	−4.9	−13.4
					G2.1 WB	0.709923 ± 18	115.2	−4.5	−13.5
					G2.2 WB	0.709935 ± 17	117.0		
					G2.3 WB	0.709984 ± 21	113.0	−6.0	−14.7
					G3.1 WD	0.709588 ± 12	49.6	−5.1	−13.9
					G3.2 WD	0.709607 ± 10	52.8		
					G3.3 WD	0.709651 ± 9	62.1		
					G3.4 WD	0.709587 ± 9	56.4	−5.5	−14.0
Friesland 7	Wijnaldum	1988	F7	28	WL	0.709199 ± 10	80.3	−6.4	−14.3
					WM	0.709206 ± 10	77.3	−6.8	−14.3
					C2.1	0.709178 ± 10	65.6	−6.6	−14.8
					G1.1 C1	0.709188 ± 10	61.1	−5.2	−14.8
					G1.2 C1	0.709181 ± 19	72.6		
					G1.4 C1	0.709185 ± 9	79.1	−5.6	−15.1
Limburg 6	Maastricht	1974	M6	48	WL	0.709351 ± 8	58.0	−6.2	−13.1
					C1.1	0.709206 ± 8	64.3	−6.0	−13.4
					G1.1 WM	0.709291 ± 10	62.5	−5.0	−13.1
					G1.2 WM	0.709296 ± 9	61.9		
					G1.3 WM	0.709285 ± 8	62.6		
					G1.4 WM	0.709268 ± 11	57.3	−5.1	−13.5
South Africa	Johannesburg		J	18	WL	0.713300 ± 7	67.6	−0.5	−9.1
					WM	0.713030 ± 10	58.5	−2.3	−9.2
					WD	0.713260 ± 10	52.9	−2.2	−9.3
					G1.1 WB	0.713280 ± 10	60.9	−0.8	−9.7
					G1.2 WB	0.713319 ± 9	58.2		
					G1.3 WB	0.713297 ± 10	56.4		
					G1.4 WB	0.713340 ± 10	61.8	−1.7	−10.0
Dominican Republic	Santo Domingo	1971	DR	18	WL	0.708310 ± 9	130.5	−4.3	−9.1
					WM	0.708301 ± 8		−4.2	−9.3
					C1.1	0.708299 ± 9	113.3	−4.5	−9.5
					C2.1	0.708309 ± 10	118.4	−4.5	−9.4
					G1.1 C3	0.708321 ± 9	107.0	−3.2	−9.3
					G1.2 C3	0.708299 ± 8	130.4		
					G1.3 C3	0.708307 ± 9	131.2		
					G1.4 C3	0.708309 ± 10	125.8	−3.8	−9.5
Somalia	unknown	1987	S	48	WB	0.707344 ± 11	301.3	−2.3	−12.3
					WD	0.707351 ± 7	315.0	−2.8	−12.1
					G1.1 WM	0.707397 ± 9	285.2	−1.8	−12.3
					G1.2 WM	0.707381 ± 9	323.6		
					G1.3 WM	0.707378 ± 9	304.8		
					G1.4 WM	0.707382 ± 10	301.1	−2.1	−12.0

*Note:* Information is provided on the geographical location (city), sample ID, dental element (using the FDI World Dental Federation notation—ISO 3950), sample location (described in Figure 2), location of caries, strontium isotope ratio (*n* = 148) and concentration (*n* = 146), and oxygen and carbon isotope values (*n* = 132).

Cusp samples (C1.1) and bulk samples were taken from the same third molar for Sr isotope analysis, as described earlier and in Table 1. The bulk enamel samples represent ~90% of the enamel of a dental element (excluding the surface enamel and the enamel from C1.1). The bulk samples ranged from 273 to 1,310 mg, of which 1–2% aliquots were taken after sample dissolution (Plomp et al., 2019).

### 2.3 Strontium isotope analysis

Strontium isotope analysis (composition and concentration) was performed on powdered enamel samples (0.8–4.8 mg, median = 1.7 mg) and aliquots (1–2%) of bulk enamel samples. To extract strontium from the enamel, samples were dissolved in 3 N HNO_3_ and chromatographic separation was performed in a class 100 clean laboratory. All PFA laboratory equipment was precleaned (Plomp et al., 2017; Plomp, Smeets, & Davies, 2020). Aliquots of an in‐house synthetic tooth standard (TSTD, 0.05 mL, 500 ng Sr, 5 mg CaHPO_4_) were used as quality control (Plomp et al., 2017). The blanks and Sr concentrations were determined by isotope dilution using an ^84^Sr spike (Plomp, Smeets, & Davies, 2020). Strontium isotope analyses were performed on a Thermo Scientific Triton *Plus* thermal ionization mass spectrometer (TIMS) using 10^11^ Ω resistors (Koornneef, Bouman, Schwieters, & Davies, 2014). Standards and 50% of the samples were loaded on out‐gassed annealed rhenium filaments in 1–2 μl 10% HNO_3_, with 1.5 μl TaCl_5_. Strontium isotope ratios were corrected to ^86^Sr/^88^Sr = 0.1194 using the exponential mass‐fractionation law. Standards measured during the study resulted in ^87^Sr/^86^Sr = 0.710247 ± 17 (*n* = 51) for NBS987 (100–200 ng) and 0.707854 ± 19 (*n* = 97) for the internal full procedure standard (TSTD). The error in the TSTD value is taken as the analytical error of the study. The procedural blanks ranged from 10.5 to 59.1 pg (*n* = 38, median = 18.4), negligible compared with typical presence of strontium in enamel (50–500 ppm, Bentley, 2006).

### 2.4 Oxygen and carbon isotope analysis

Oxygen and carbon isotope analysis was performed on powdered enamel. The sample (0.3–0.8 mg) was weighed into an exetainer vial. The prepared vials were placed in a sample block interspaced with calibration and control standards VICS and IAEA‐603. After flushing the vials with helium, samples and standards were acidified with water‐free H_3_PO_4_ (100%) at 45°C and allowed to react for 24 hr. The gas mixture was analyzed using a Thermo Finnigan Delta plus IRMS with a GasBench II. The isotopic values are reported as *δ* (delta) values in ‰ units. Values were normalized to international standard IAEA‐603 (δ^18^O = −2.6 ± 0.17 and δ^13^C = 2.5 ± 0.04 [1*σ*, *n* = 7]) and are reported relative to the Vienna Peedee Belemnite (VPDB) standard.

### 2.5 Statistical analyses

Statistical assessments were performed using GraphPad Prism7. Data were examined for normality using D'Agostino & Pearson normality test (see Plomp, Verdegaal‐Warmerdam, & Davies, 2020 for details). Statistical significance used for the ultrahigh‐density analyses (*n* = 20) of dental elements T1.1, T2.1, and T6.1 was determined using one‐way ANOVA, followed by Tukey's multiple comparisons post hoc analysis. To facilitate comparisons between results, differences between the lowest and highest isotopic results are indicated (Δ_max−min_), as well as the average isotopic intraindividual variation, or avg_isovar (Equation 1, where *n* is the number of individuals analyzed and where 2*σ* is taken for strontium isotope and 1*σ* for oxygen and carbon isotope analyses).

(1) $$a\mathrm{vg}\_\text{isovar}=\sqrt{\frac{1}{n}*\sum_{i=1}^{n}\sigma_{i}^{2}}$$

## 3 RESULTS

To evaluate the intraindividual variation in Sr–O–C isotopes in modern human enamel, the results of the multiloci sampling approach (Table 2) have been organized based on the density of the sampling approach. First, the results of the ultrahigh‐density samples are presented (Section 3.1), followed by the high‐density samples (Section 3.2), and single cusp location versus bulk sampling approaches (Section 3.3). Finally, the high‐density sampling results from dental elements affected by caries are outlined (Section 3.4).

### 3.1 Intradental variation indicated by ultrahigh‐density sampling

**FIGURE 3:**
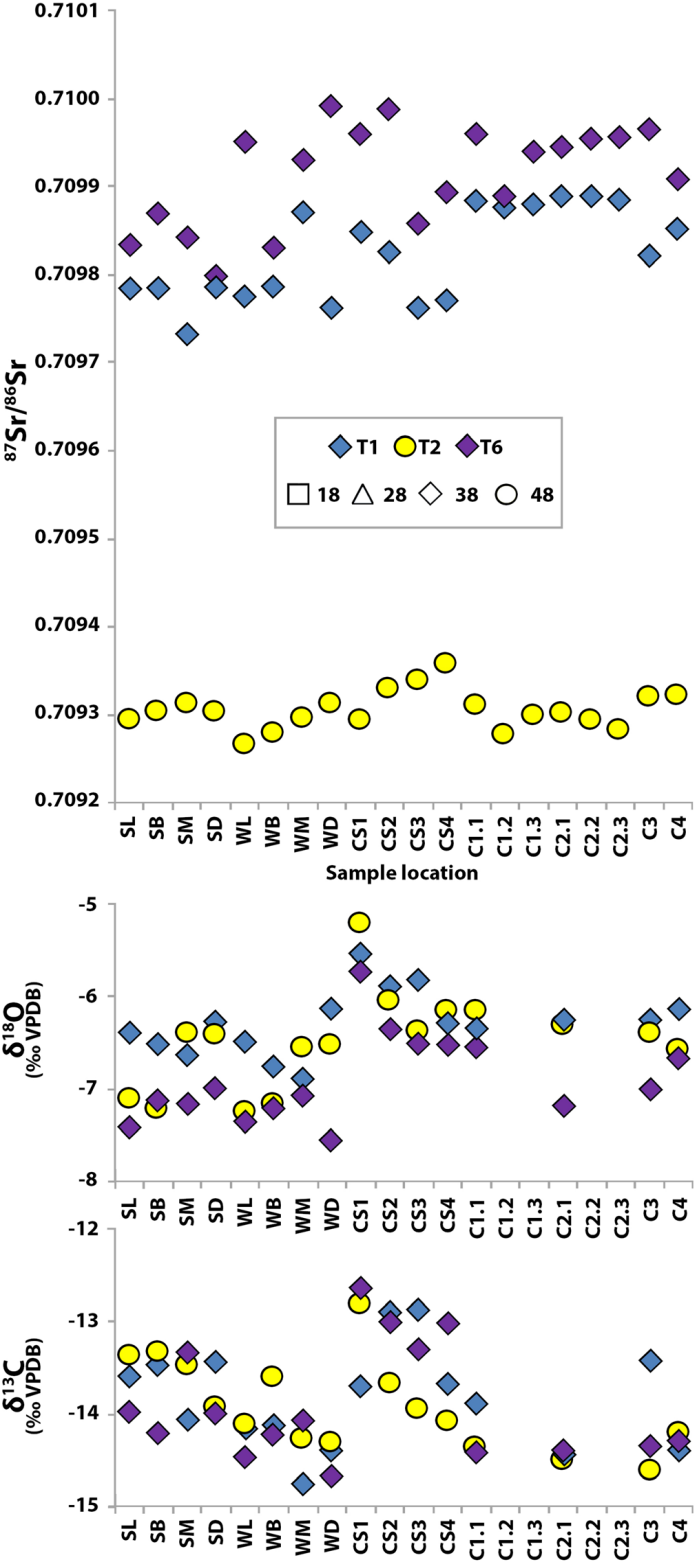
Strontium, oxygen, and carbon isotope data for individuals T1 (blue), T2 (yellow), and T6 (purple). Dental elements are indicated by markers using the FDI World Dental Federation notation (ISO 3950): square = upper right M3 (18), triangle = upper left M3 (28), diamond = lower left M3 (38), and circle = lower right M3 (48). The *x*‐axis indicates sampling location (see Figure 2 and Table 1)

The Sr–O–C isotope results for the three individuals (T1, T2, T6) sampled using the ultrahigh‐density approach are shown in Figure 3. Differences in strontium isotope ratios (Δ^87^Sr/^86^Sr_max‐min_) ranged from 0.000091 to 0.000193 (up to 10 times larger than the analytical error) (Table 3). Sr concentrations appear randomly distributed with the largest spread in Sr concentrations found in T1.1 (Δ Sr ppm_max−min_ = 36.6 ppm, Table 3). Variation in δ^18^O and δ^13^C isotope values ranged between 1.3‰ and 2.0‰ (up to 12 times the analytical error for δ^18^O and 50 times higher for δ^13^C) (Table 3).

**TABLE 3:** Strontium, oxygen, and carbon isotope results for the individuals from the Netherlands, Johannesburg, Dominican Republic, and Somalia (see Table 2 for sample IDs)

	^87^Sr/^86^Sr						Sr ppm						δ^18^O (‰ VPDB*)*					δ^13^C (‰ VPDB)					
	Min	Max	Δ_(max−min)_	Median	2 *SD*	*n*	Min	Max	Δ_(max−min)_	Median	2 *SD*	*n*	Min	Max	Δ_(max−min)_	Median	2 *SD*	Min	Max	Δ_(max−min)_	Median	2 *SD*	*n*
T1 (T1.1, T1.2, T1.3)	0.709732	0.709908	0.000176	0.709862	±102	32	33.9	78.8	44.9	52.6	14.3	32	−6.9	−5.0	1.9	−6.1	0.8	−14.9	−12.8	2.1	−14.0	1.3	36
T1.1	0.709732	0.709890	0.000157	0.709823	±105	20	42.3	78.8	36.6	52.3	14.8	20	−6.9	−5.5	1.3	−6.3	0.7	−14.8	−12.9	1.9	−13.8	1.1	16
T1.1	0.709762	0.709884	0.000122	0.709804	±102	6	54.0	78.8	24.8	56.0	18.9	6	−6.9	−6.1	0.7	−6.4	0.6	−14.8	−13.4	1.3	−14.1	0.9	6
T1.2	0.709802	0.709900	0.000098	0.709872	±70	6	48.5	55.9	7.4	51.3	5.6	6	−6.7	−5.8	0.9	−5.9	0.8	−14.6	−13.9	0.6	−14.4	0.6	6
T1.3	0.709864	0.709908	0.000044	0.709884	±33	6	33.9	60.3	26.3	55.7	20.0	6	−6.2	−5.8	0.4	−6.0	0.3	−14.9	−14.1	0.8	−14.5	0.5	7
T2 (T2.1, T2.2)	0.709240	0.709357	0.000117	0.709302	±48	26	31.1	43.8	12.7	38.1	4.7	26	−7.2	−5.2	2.0	−6.4	0.9	−14.7	−12.8	1.9	−14.2	1.0	23
T2.1	0.709266	0.709357	0.000091	0.709302	±44	20	31.1	43.8	12.7	37.7	4.5	6	−7.2	−5.2	2.0	−6.4	1.0	−14.6	−12.8	1.8	−14.0	1.0	16
T2.1	0.709266	0.709321	0.000055	0.709303	±43	6	36.5	39.1	2.6	38.2	1.8	6	−7.2	−6.2	1.1	−6.5	0.9	−14.6	−13.6	1.0	−14.3	0.7	6
T2.2	0.709240	0.709323	0.000083	0.709299	±61	6	38.7	42.7	4.0	39.6	3.3	6	−7.0	−5.9	1.0	−6.5	0.7	−14.7	−14.2	0.5	−14.4	0.3	7
T6 (T6.1, T6.2)	0.709799	0.709991	0.000192	0.709891	±130	26	55.8	88.4	32.6	68.7	18.5	26	−7.6	−5.7	1.8	−6.8	1.0	−14.7	−12.7	2.0	−14.1	1.1	23
T6.1	0.709799	0.709991	0.000192	0.709935	±115	20	55.8	77.5	21.7	65.7	12.8	20	−7.6	−5.7	1.8	−7.0	1.2	−14.7	−12.6	2.0	−14.1	1.2	16
T6.1	0.709830	0.709991	0.000161	0.709956	±113	6	61.4	74.8	13.4	66.5	11.8	6	−7.6	−6.5	1.0	−7.1	0.7	−14.7	−14.1	0.6	−14.4	0.4	6
T6.2	0.709799	0.709835	0.000036	0.709820	±27	6	78.2	88.4	10.2	82.1	8.7	6	−6.9	−6.0	0.9	−6.5	0.7	−14.4	−13.7	0.6	−14.0	0.4	7
D3	0.709539	0.709600	0.000061	0.709567	±45	6	87.3	103.3	16.0	93.7	10.7	6	−6.9	−5.5	1.4	−6.2	0.9	−12.9	−12.6	0.3	−12.8	0.3	6
D13	0.709202	0.709223	0.000021	0.709209	±16	6	94.6	100.6	6.0	96.2	4.6	5	−6.3	−5.9	0.4	−6.1	0.3	−14.0	−13.6	0.4	−13.9	0.3	6
D15	0.709383	0.709412	0.000029	0.709394	±21	6	56.8	100.6	43.8	62.5	33.1	6	−6.6	−5.9	0.7	−6.1	0.6	−14.0	‐13.5	0.5	−13.7	0.3	6
*Dutch Caries*																							
F6	0.709535	0.709984	0.000449	0.709588	±319	13	42.3	117.0	74.7	54.3	55.8	13	−6.2	−4.5	1.7	−5.5	1.1	−14.7	−13.4	1.3	−13.8	0.8	8
F7	0.709178	0.709206	0.000029	0.709187	±22	6	61.1	80.3	19.2	74.9	15.6	6	−6.8	−5.2	1.6	−6.4	1.3	−15.1	−14.3	0.8	−14.8	0.7	5
M6	0.709206	0.709351	0.000145	0.709288	±94	6	57.3	64.3	7.0	62.2	5.6	6	−6.2	−5.0	1.2	−5.6	1.3	−13.5	−13.1	0.4	−13.2	0.4	4
*Non−Dutch Caries*																							
J	0.713030	0.713340	0.000309	0.713297	±210	7	52.9	67.6	14.8	58.5	9.3	7	−2.3	−0.5	1.8	−1.7	1.6	−10.0	−9.1	0.9	−9.3	0.8	5
DR	0.708299	0.708321	0.000022	0.708308	±15	8	107.0	131.2	24.3	125.8	19.3	7	−4.5	−3.2	1.3	−4.2	1.0	−9.5	−9.1	0.5	−9.3	0.3	6
S	0.707344	0.707397	0.000053	0.707380	±41	6	285.2	323.6	38.4	303.1	26.3	6	−2.8	−1.8	1.0	−2.2	0.8	−12.3	−12.0	0.3	−12.2	0.3	4

*Note:* T1, T2, and T6 represent the combined results of all dental elements of these individuals analyzed (e.g., T1 includes the combined results of T1.1, T1.2, and T1.3). The subset of six locations for T1.1, T2.1, and T6.1 are WL, WB, WM, WD, C1.1, and C3 (see Table 1 for abbreviations) to match the locations with the other sampled teeth. Dental elements F6, F7, M6, J, DR, and S were affected by caries. Individuals J, DR, and S are non‐Dutch.

There were no indications of linear correlation between enamel sample locations of T1.1, T2.1, and T6.1 and isotope system (^87^Sr/^86^Sr *R* ^2^ < 0.4, δ^18^O *R* ^2^ < 0.2, and δ^13^C *R* ^2^ < 0.4). Intradental variation between surface and inner enamel was recorded for ^87^Sr/^86^Sr, δ^18^O, and δ^13^C in T1.1 (*p* < .01) and for both ^87^Sr/^86^Sr (*p* < .04) and δ^13^C (*p* < .01) for T2.1 and T6.1. No consistent differences were found between the cusp and wall regions (with the exception of a significant difference for ^87^Sr/^86^Sr in T1.1: *p* = .01) (Plomp, Verdegaal‐Warmerdam, & Davies, 2020).

#### 3.1.1 Interdental variation: Comparison of ultrahigh and high‐density sampling methods

**FIGURE 4:**
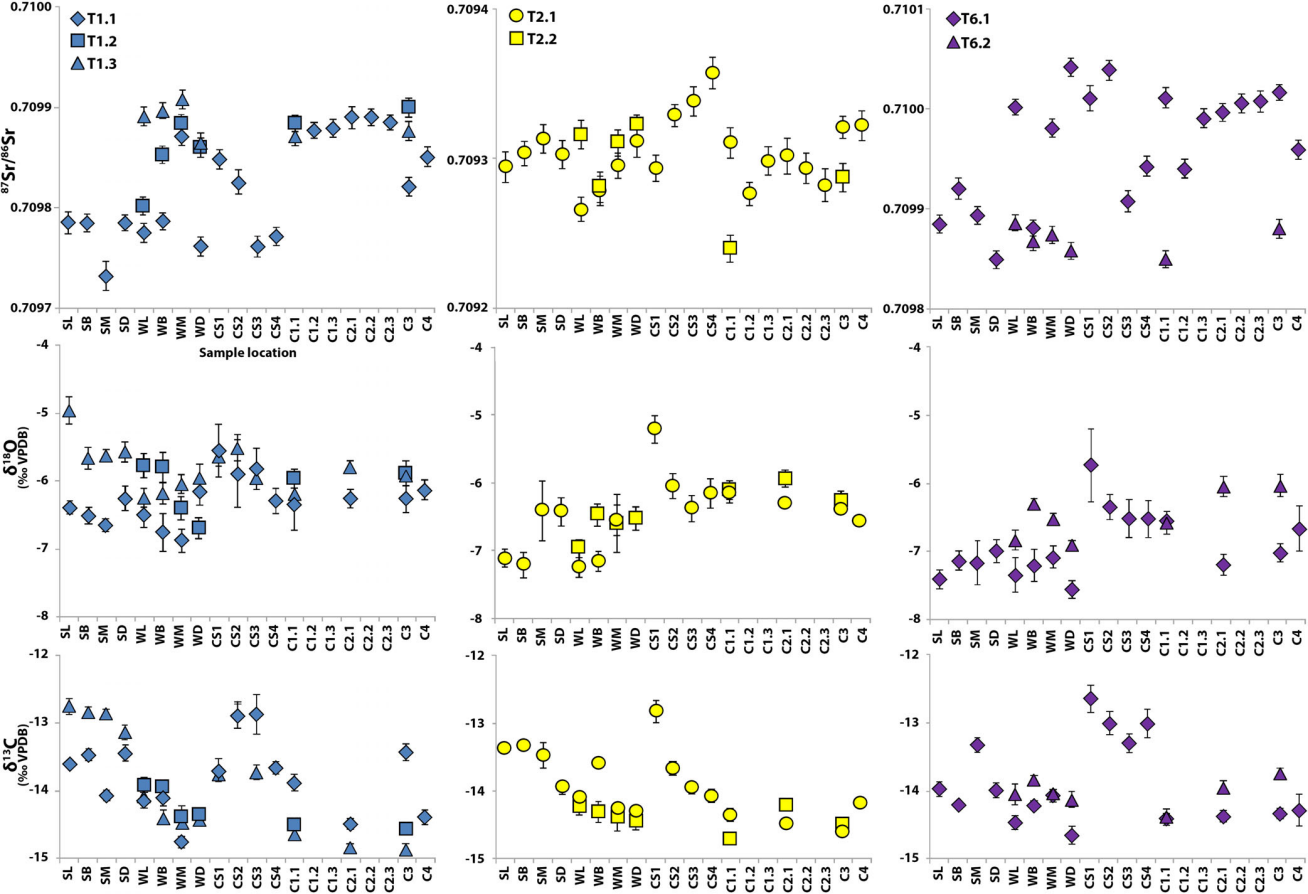
Interdental Sr–O–C isotope variation of T1, T2 and T6. The x‐axis represents the sample locations as described in Figure 2

Additional third molars were sampled from the individuals sampled with the ultrahigh‐density sampling approach (T1, T2, and T6) using the high‐density sampling approach, allowing the examination of intraindividual interdental Sr–O–C isotope variation (Figure 4). An increase in variation in Δ(_max−min_) Sr isotope ratios (0.000117–0.000192) and Sr concentrations (13–45 ppm), and oxygen and carbon isotope values (1.8–2.1‰), is seen when multiple third molars from the same individual are combined (Table 3). Interdental variation was significant for strontium (*p* < 0.02) and oxygen (*p* < .04) isotopes in T1 and T6 (T1.1 and T1.3; T6.1 and T6.2, respectively), as well as carbon isotopes in T2 (T2.1 and T2.2, *p* = .02). No consistent differences were found with respect to isotopic system, location, or dental element sampled (see Plomp, Verdegaal‐Warmerdam, & Davies, 2020 for more details).

### 3.2 Population variation indicated by high‐density sampling

**FIGURE 5:**
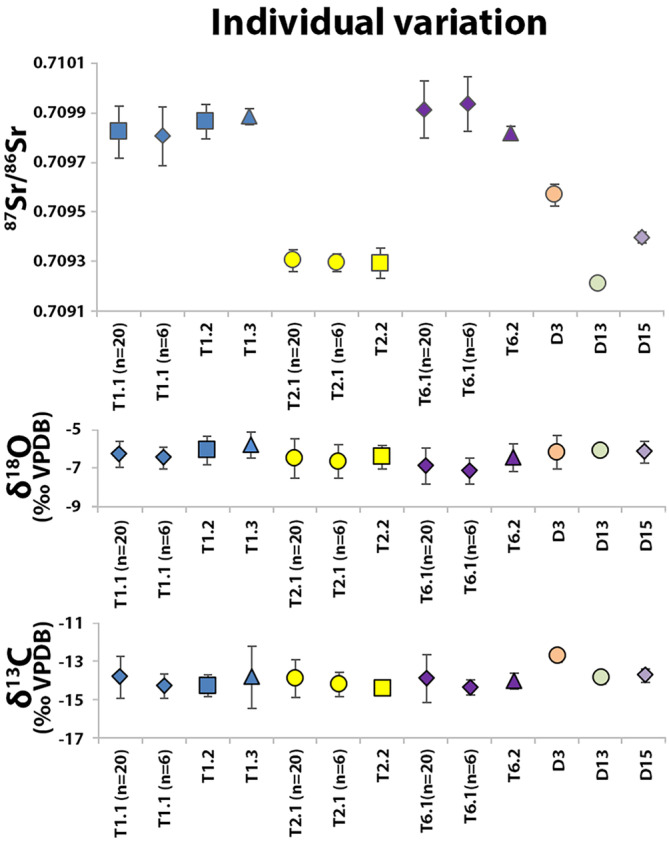
Isotopic variation plotted for each dental element per Dutch individual sampled using the (ultra)high‐density approach. The high‐density subset (*n* = 6) of T1.1, T2.1, and T6.1 consists of the same sampling locations as the other individuals sampled using the high‐density sampling approach (WL, WB, WM, WD and C1.1, C2.1)

To compare the variation seen in Sr–O–C isotopes between individuals (interindividual variation), the high‐density sampling approach was applied to six individuals from the Netherlands (T1, T2, T6, D3, D13, D15; 10 teeth in total) (Figure 5, Table 3).

#### 3.2.1 High‐density sampling across the Netherlands

The difference in ^87^Sr/^86^Sr (Δ_max−min_) within the six Dutch individuals using the high‐density‐sampling approach on inner enamel ranged from 0.000021 to 0.000161 (2 *SD* ± 16–115, avg_isovar ± 62, Table 3). Differences in Sr concentrations (ΔSr ppm_max−min_) ranged between 3 and 44 ppm (avg_isovar ± 15 ppm, Table 3). Variation seen in Δδ^18^O_max−min_ ranged from 0.3‰ to 1.4‰, and Δδ^13^C_max−min_ from 0.3‰ to 1.3‰ (avg_isovar for δ^18^O and δ^13^C ± 0.7‰, Tables 2 and 3). The average Dutch isotopic variation (avg_isovar) is three times larger than the analytical error for ^87^Sr/^86^Sr, four times for δ^18^O and 18 times for δ^13^C. The maximal differences in the inner enamel of a single dental element found in this study are ^87^Sr/^86^Sr = 0.000161, 44 ppm Sr, δ^18^O, and δ^13^C ~1.4‰, with eight times larger values seen compared with the analytical error for ^87^Sr/^86^Sr and δ^18^O and 33 times larger for δ^13^C.

#### 3.2.2 Effect of the globalized supermarket diet

The ^87^Sr/^86^Sr variation seen in inner enamel from the late 20th‐century Dutch individuals ranged from 0.709240 to 0.709991 (Δ = 0.000751, with Δ^87^Sr/^86^Sr_max−min_ = 0.000036–0.000122, 2 *SD* ± 43–113), with Sr concentrations ranging between 37 and 88 ppm. Variation is also seen in the Sr isotope data from the mid‐20th‐century individuals (Δ = 0.000398, with Δ^87^Sr/^86^Sr_max−min_ = 0.000021–0.000061, 2 *SD* ± 21–45, and Sr concentrations ranging between 57 and 103 ppm). In late 20th century enamel δ^18^O ranged from −7.6 to −5.8 (Δ = 1.8‰, 2 *SD* ± 0.6–0.9) and δ^13^C ranged between −14.8 and −13.4 (Δ = 1.4‰, 2 *SD* ± 0.4–0.9) (Tables 2 and 3). In earlier material, δ^18^O ranged from −6.9 to −5.5 (Δ = 1.4‰, 2 *SD* ± 0.3–0.9) and δ^13^C ranged between −14.0 and −12.6 (Δ = 1.4‰, 2 *SD* ± 0.3–0.5) (Table 3).

### 3.3 Single locus cusp versus bulk sampling

**FIGURE 6:**
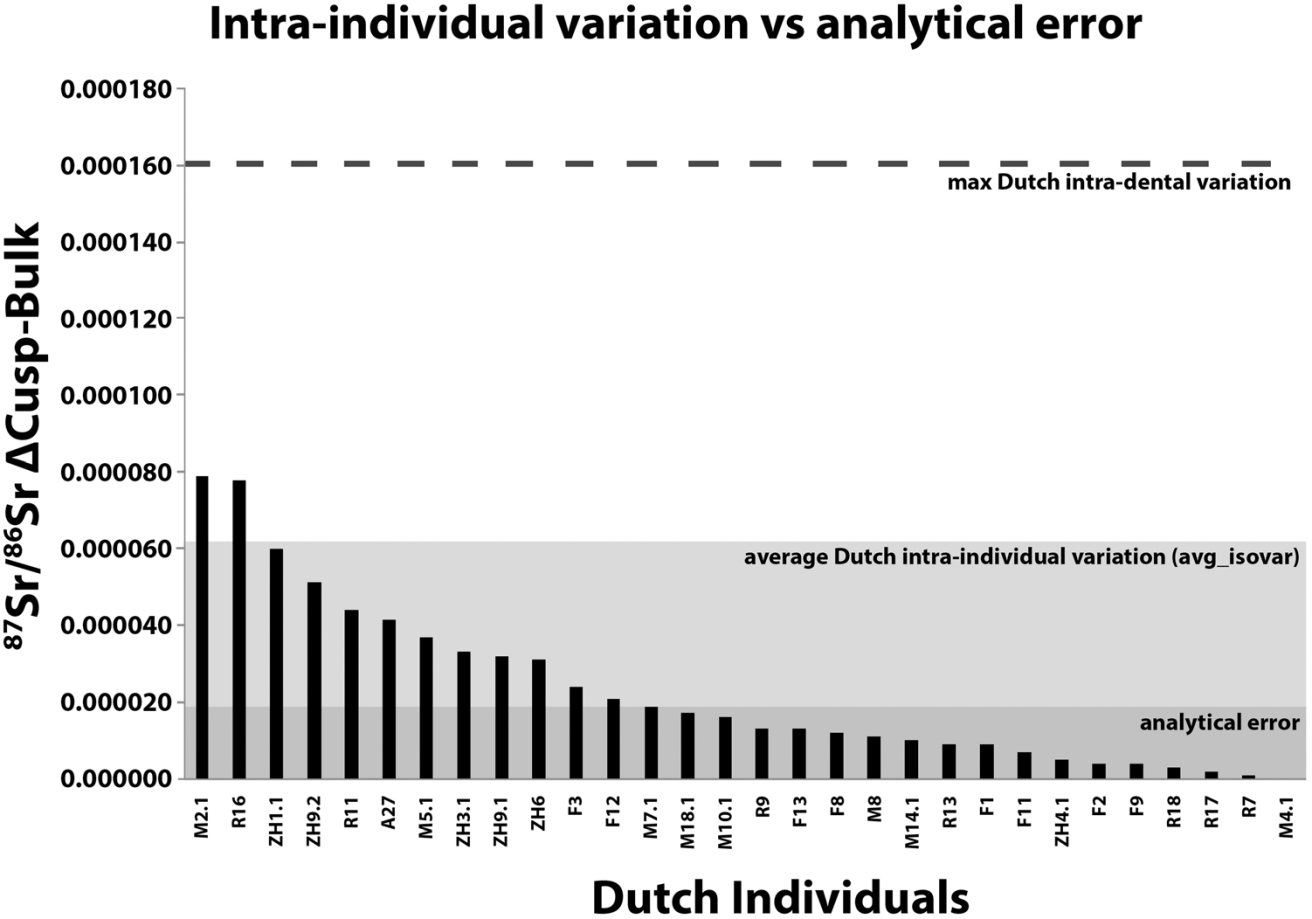
The difference in Sr isotope ratio recorded between bulk and cusp samples from an individual. Note that the analytical error does not accurately capture the intraindividual variation seen in Sr isotope composition of Dutch individuals; see Table 4 for all ΔCusp–Bulk values

The ^87^Sr/^86^Sr ratios of the cusp and bulk samples of the same individual were within analytical error (Δ^87^Sr/^86^Sr_Bulk‐Cusp_ 2 *SD* ± 19) in only 60% of the cases (18 out of 30 individuals; Table 4, Figure 6). When the average Dutch intraindividual variation is taken (avg_isovar ± 62), C1.1 is representative for the bulk isotopic results in 93.3% of the individuals (*n* = 28) (Figure 6). The variation in ^87^Sr/^86^Sr seen in non‐Dutch dental elements (*n* = 6) was similar, ranging from Δ_Cusp‐Bulk_ = 0.000020–0.000080.

**TABLE 4:** Strontium composition analyses from cusp samples (^87^Sr/^86^Sr Cusp) and bulk analyses (^87^Sr/^86^Sr Bulk) of the same individual (*n* = 35), with differences between Cusp and Bulk analyses in the last column (Δ Cusp−Bulk) and Figure 6

Individual	City	Birth year	^87^Sr/^86^Sr Cusp	2 *SE*	^87^Sr/^86^Sr Bulk	2 *SE*	Δ_Cusp−Bulk_
A27	Alkmaar	1984	0.709409	±8	0.709367^a^	±7	0.000042
R7	Dordrecht	1989	0.708854	±8	0.708853	±9	0.000001
R9	Rotterdam	1992	0.709808	±8	0.709821	±9	0.000013
R11	Rotterdam	1987	0.709331	±6	0.709375^a^ ^,^ ^b^	±11	0.000044
R13	Rotterdam	1979	0.709418	±9	0.709409^a^ ^,^ ^b^	±9	0.000009
R16	Dordrecht	1996	0.709188	±8	0.709266	±6	0.000078
R17	Rotterdam	1998	0.709585	±6	0.709583	±7	0.000002
R18	Rotterdam	1999	0.709356	±6	0.709359	±7	0.000003
F1	Lippenhuizen	1994	0.709423	±8	0.709432^a^ ^,^ ^b^	±9	0.000009
F2	Leeuwarden	1995	0.709303	±10	0.709307	±7	0.000004
F3	Holwerd	1998	0.709643	±8	0.709619^a^	±9	0.000024
F8	Leeuwarden	1992	0.709458	±8	0.709469^a^	±8	0.000012
F9	Leeuwarden	1985	0.709386	±9	0.709390	±9	0.000004
F11	Leeuwarden	1993	0.709237	±9	0.709230^a^	±9	0.000007
F12	Oldeboorn	1992	0.709142	±9	0.709122^a^	±9	0.000021
F13	Leeuwarden	1976	0.709325	±10	0.709337^a^	±9	0.000013
ZH1.1	Heerlen	1998	0.709364	±10	0.709424^a^ ^,^ ^b^	±8	0.000060
ZH3.1	Heerlen	1992	0.709352	±9	0.709319^a^ ^,^ ^b^	±9	0.000033
ZH4.1	Heerlen	1997	0.709175	±9	0.709169^a^ ^,^ ^b^	±9	0.000005
ZH6	Heerlen	1987	0.708869	±11	0.708838	±10	0.000031
ZH9.1	Heerlen	1998	0.709830	±9	0.709862^a^	±8	0.000032
ZH9.2	Heerlen	1998	0.709565	±8	0.709616	±10	0.000051
M2.1	Maastricht	1972	0.709217	±9	0.709296	±10	0.000079
M4.1	Maastricht	1983	0.709596	±9	0.709596^a^	±10	0.000000
M5.1	Maastricht	1996	0.709608	±9	0.709644^a^	±10	0.000037
M7.1	Maastricht	1995	0.709792	±8	0.709811	±10	0.000019
M8	Maastricht	1990	0.709363	±8	0.709352	±9	0.000011
M10.1	Maastricht	1993	0.709518	±11	0.709502	±9	0.000016
M14.1	Maastricht	1990	0.709536	±9	0.709546^a^ ^,^ ^b^	±10	0.000010
M18.1	Maastricht	1994	0.709327	±7	0.709310	±9	0.000017
W2‐R8	Curaçao (Willemstad)	NA	0.709445	±7	0.709375^a^	±10	0.000070
W3‐B4	Bonaire (Kralendijk)	1983	0.709296	±7	0.709256^a^ ^,^ ^b^	±9	0.000040
W4‐B16	Columbia (Cúcuta)	NA	0.711767	±9	0.711749^a^ ^,^ ^b^	±9	0.000018
W5‐I	Iceland (Reykjavik)	NA	0.708721	±10	0.708740^a^	±9	0.000019
W6‐B13	Bonaire/Aruba/Curaçao	1999	0.709584	±9	0.709504	±10	0.000080
W7‐Am	United States (Massachusetts)	NA	0.709258	±8	0.709218	±9	0.000041

a Previously published in Plomp et al. (2019).

b Bulk sample consistent of two‐third molars.

### 3.4 The effect of caries on isotopic composition

**FIGURE 7:**
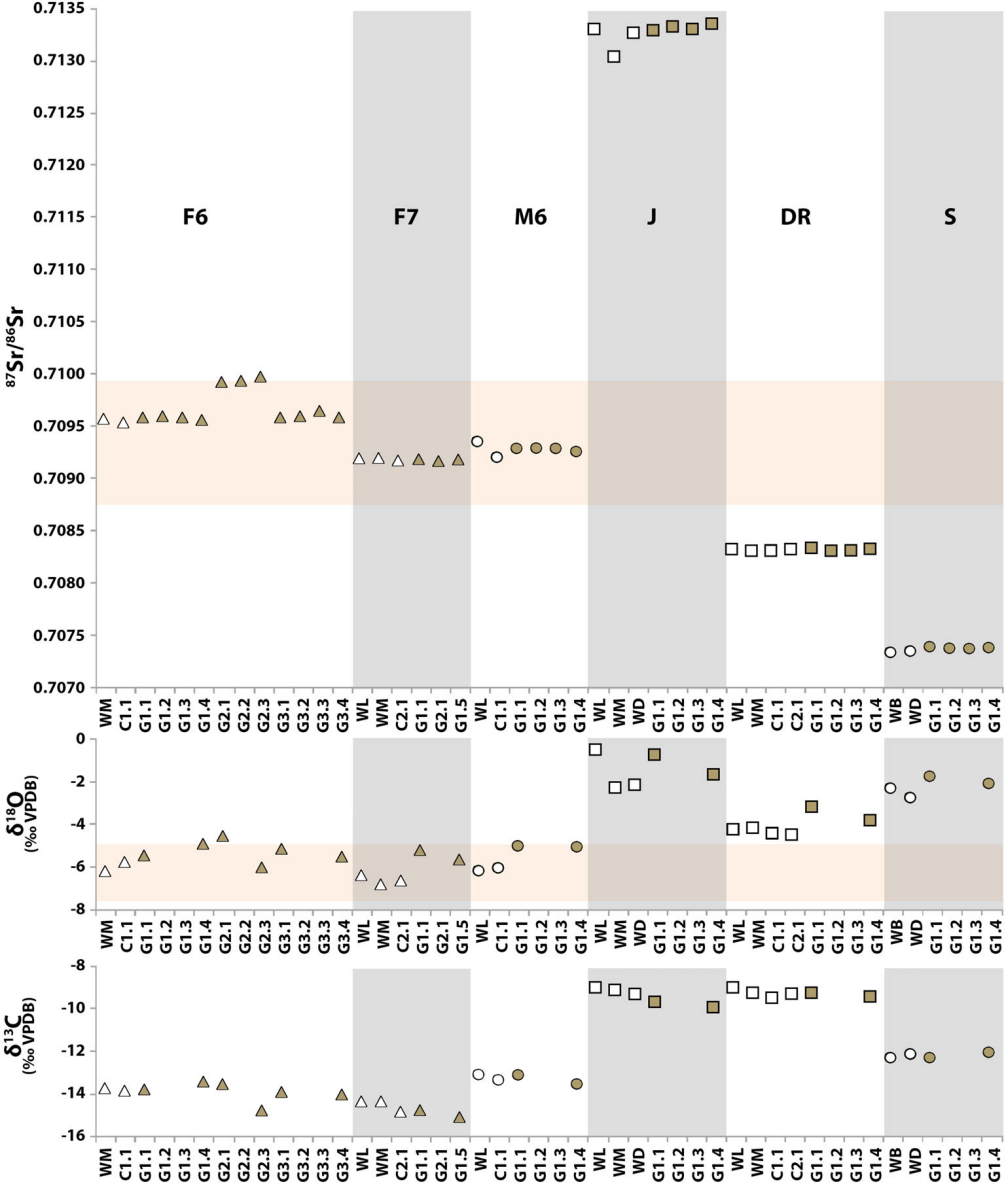
Sr–O–C isotope variation seen in dental elements affected by caries. Unaffected enamel in white, carious enamel in brown. The orange area represents the Dutch range based on human enamel from Dutch residents reported in this study (^87^Sr/^86^Sr = 0.7088–0.7099, δ^18^O = −7.6‰ to −5.0‰)

To evaluate the effect of caries on the isotopic composition of dental enamel, both unaffected and affected locations were sampled from the same dental element (Table 3, individuals F6, F7, M6, J, DR, and S; Figure 7). The Sr isotope ratios of carious enamel are generally within error of unaffected enamel (Figure 7), with Δ^87^Sr/^86^Sr_max–min_ ranging from 0.000022 to 0.000449 (avg_isovar ± 162) and ΔSr ppm _max−min_ ranging from 7.0 to 74.7 ppm (avg_isovar ± 28). Variation seen in δ^18^O Δ_max−min_ ranged from 1.0‰ to 1.8‰, and Δδ^13^C_max‐min_ from 0.3‰ to 1.3‰ (avg_isovar for δ^18^O = 1.2‰ and δ^13^C ± 0.6‰, Table 3). Elevated δ^18^O values were found in caries for all six individuals, with significant differences (*p* = .01) in F7, M6, and DR. Unaffected and carious enamel showed similar δ^13^C and ^87^Sr/^86^Sr values (Table 3, Figure 7), with the exception (*n* = 1 out of 8) of the buccal caries in F6 (G2), which showed elevated Sr values (Δ^87^Sr/^86^Sr = 0.0004) as well as higher Sr concentrations (ΔSr ppm = 64). Despite these isotopic variations in Sr and O, all the data from the Dutch individuals was compatible with the Dutch Sr–O isotope range (Figure 7).

## 4 DISCUSSION

The maximum intraindividual variation in inner dental enamel seen in this study (^87^Sr/^86^Sr = 0.000161, 44 ppm Sr, δ^18^O and δ^13^C ~1.4‰) highlights the importance of quantifying intraindividual isotopic variation before interpretations on mobility and diet are made.

### 4.1 Intra‐ and interindividual variation

Previous studies proposed possible diagenetic Sr contributions to surface enamel (Dufour et al., 2007; Horn & Müller‐Sohnius, 1999). Differences in Sr ratios in surface and inner enamel were present in all three individuals (T1, T2, T6) examined using the ultrahigh‐density sampling approach (Section 3.1). Nevertheless, inner enamel of both wall and cusp regions in the high‐density samples (excluding surface enamel) was representative of the results of the ultrahigh‐density (including surface samples) Sr–O–C isotope results of the same third molar (Table 3). Omitting the surface measurements decreased the total Sr isotope variation by 20% (^87^Sr/^86^Sr_all_ 2 *SD* ± 40–120 to ^87^Sr/^86^Sr_inner_ 2 *SD* ± 40–100). The median ^87^Sr/^86^Sr results for individuals are, however, essentially unchanged when surface measurements are excluded (with the exception of T1.1 where Δ^87^Sr/^86^Sr_median[all‐inner]_ = 0.000050, three times the analytical error). Future research involving more individuals sampled (*n* > 20) with a similar number of sample loci (*n* > 6) is required to provide conclusive evidence of Sr contamination of enamel surface. Such results would be particularly relevant for unburied dental elements in forensic cases.

Differences between surface and inner enamel were also present in δ^13^C for all three individuals (T1, T2, T6) analyzed using the ultra‐high sampling approach and for one individual (T1) in δ^18^O. When omitting the surface measurements, the oxygen and carbon variation decreased by 15–25% for δ^18^O and 25–60% for δ^13^C (δ^18^O 2 *SD*_total_ = 0.7–1.2 compared with δ^18^O 2 *SD*_inner_ = 0.6–0.9 and δ^13^C 2 *SD*_total_ = 1.0–1.2 compared with δ^13^C 2 *SD*_inner_ = 0.4–0.9), with lower median differences (0.6–1.0‰ for δ^18^O and 0.3‰ for δ^13^C) in the inner enamel. Omitting the surface measurements has a greater effect on the median O and C isotope results in comparison with Sr (up to six times the analytical error for oxygen and eight times for carbon). Significantly elevated δ^13^C values are found in surface enamel (*p* < .02) (Figure 3). Greater variability in C isotope values might be explained by differential isotopic incorporation in surface enamel or possible surface exchange. A change in diet at the time of the formation of the surface enamel, which is secreted later than the inner enamel (Piesco & Avery, 2002), may also explain the difference seen in surface and inner enamel seen in carbon isotopes. As the surface cusp samples (CS1, CS2, CS3, and CS4) are taken from the same incremental enamel layer, this could indicate that incremental isotopic signatures are retained instead of being averaged during the mineralization phase. Nevertheless, there is large variation seen in the cusp surface samples (Figure 2). Furthermore, a potential temporal influence is difficult to assess for the current study as the formation of the dental elements was not recorded in such detail, most of the samples do not represent restricted time periods in enamel formation, and specific dietary information during the enamel formation is unavailable. These data do, however, suggest that surface enamel sampling should be avoided for carbon isotope analysis until incorporation of δ^13^C values in surface enamel is better understood.

The ultrahigh‐density sampling approach showed limited intradental variation within inner enamel, with no differences recorded between the inner cusp and wall regions (with the exception of elevated ^87^Sr/^86^Sr in the cusp samples of T1.1). This indicates that the currently preferred sampling regions (buccal/lingual tooth wall) can be extended to include cusp regions (where attrition allows occlusal sampling), as these regions of inner enamel are expected to give comparable results.

Comparison of cusp and bulk Sr isotope analyses indicates that a single inner enamel cusp measurement (C1.1) of Dutch third molars is representative of the bulk enamel value in only 60% of the dental elements analyzed (Figure 6). The two samples with the largest differences in ^87^Sr/^86^Sr in cusp and bulk samples (0.000078/9) belong to individuals M2.1 and R16, raised in a single location (Maastricht and Dordrecht, respectively), with individual R16 indicating that they did not consume fish. These data demonstrate that even where individuals are sedentary, the dental Sr isotope ratios are heterogeneous. Although the current study did not contrast single versus bulk samples for oxygen and carbon isotopes, results for oxygen and carbon isotopes in caprid (*Ammotragus lervia*, Reade et al., 2015) similarly suggest that single location samples may not represent the average isotopic value of the dental element, and that bulk sampling condenses the full isotopic variation within a dental element.

Future studies are encouraged select their sampling strategies based on the type of isotopic variation they would like to evaluate. In cases where intraindividual variation should be assessed, or when the life history of an individual is examined, the multiloci or high‐density approach is more effective than single loci sampling. If interindividual variation is determined and only an estimation of the isotopic signature is required, single loci sampling may be sufficient. As bulk sampling averages the total isotopic variation and requires large sample sizes, this method may not be applicable. Compared with bulk analyses, single‐loci sampling approaches provide similar information, are more efficient, and are less destructive. In situ sampling analysis using laser ablation inductively coupled mass spectrometry (LA‐ICP‐MS) analysis may be better suited to assess the intraindividual variation in Sr concentration and composition in a dental element (Smith et al., 2018; Willmes et al., 2016).

The Sr–O–C isotope results from Dutch individuals in this study contribute to the already established Dutch isotopic ranges based on modern enamel for strontium and oxygen and provide an indication of the Dutch carbon isotope range. The ^87^Sr/^86^Sr results of this study (0.708838–0.709991) confirm ranges reported in previous studies (0.7078–0.7099, Font et al., 2015; Plomp et al., 2019). The δ^18^O values in the current study range from −7.6‰ to −5.0‰ (Δ = 2.6‰), confirm results of a previous study reporting a wider range (−7.6‰ to −4.5‰, Δ = 3.1‰, Font et al., 2015), as well as previous estimations for archeological populations of 2–3‰ (Lightfoot & O'Connell, 2016; Wright, 2013). Modern Dutch enamel δ^13^C values in the current study range from −14.9‰ to −12.6‰ (Δ = 2.3‰).

The (ultra)high‐density sampling approach showed considerable intraindividual isotopic variation within modern Dutch individuals. Significant interdental variation was seen in strontium and oxygen isotopes in two out of three individuals (T1 and T6), indicating that a single third molar is not always representative of the isotopic results of other third molars and possibly other dental elements. Using the high‐density sampling approach, the maximum differences in isotopic results from inner enamel of a single dental element reached 0.000161 for strontium, 1.4‰ for oxygen, and up to 1.3‰ for carbon. Increased variation is seen using the ultrahigh‐density sampling approach (including results from additional molars from the same individual), with maximum intraindividual variation (^87^Sr/^86^Sr = 0.000192, ~2‰ for oxygen and carbon, Table 3) approaching levels of variability in the Dutch population for oxygen (δ^18^O = 3.1‰, Font et al., 2015) and carbon (δ^13^C = 2.3‰). Intraindividual Sr variation did not reach interindividual/population variation, which was 10 times higher (Δ = ~0.002). The analytical precision (^87^Sr/^86^Sr ± 0.000019, δ^18^O ± 0.17‰ and δ^13^C ± 0.04‰) is therefore significantly less than the intraindividual and population variability recorded here. The estimated variation based on the average Dutch isotopic intraindividual variation (avg_isovar) of the inner enamel of single dental elements in this study (^87^Sr/^86^Sr ± 0.000062, Sr ppm ± 15, δ^18^O and δ^13^C ± 0.7‰) provides estimations for the expected variation in modern Dutch third molars. The maximum intraindividual differences seen in this study for inner enamel (^87^Sr/^86^Sr = 0.000161, Sr ppm = 44, δ^18^O = 1.4‰ δ^13^C = 1.3‰) can be used as an indication for the maximal expected variation within inner enamel of a dental element and may increase in future studies when more individuals are analyzed using a high‐density sampling approach. Third molars are the most variable dental element in the human dentition in terms of enamel formation and eruption (AlQahtani et al., 2010; Reid & Dean, 2006), and they can therefore be expected to record the largest isotopic variation in the dentition of habitual diet and residence. Consequently, studies using other dental elements may be expected to exhibit less isotopic variation as time frames of dental development are shorter and more constrained. In order to establish how representative this study is of the isotopic variability recorded in human dentition, it would be of particular interest to study first/second molars and pre‐molars, as these are most often sampled in archeological/forensic provenance studies. A comparative study of archeological inner enamel of dental elements would also be of interest, keeping in mind the effects that diagenesis may have on the isotopic composition of archeological dental elements.

### 4.2 Temporal and spatial effects on Sr–O–C isotope variation

Previous studies (Chesson et al., 2011; Valenzuela et al., 2012; Vautour et al., 2015) have highlighted the potential influence of the modern global supermarket diet on the isotopic results of modern human tissues. Assessing the impact of globalization on the individuals in this study is complicated as the individuals originate from various areas in the Netherlands, which will likely result in some degree of isotopic variation due to different sources of potable water and variation in local food availability. In addition, variation is expected due to different ages of the individuals. Most importantly, only three individuals representative of the pre‐supermarket era were sampled. More samples from individuals born before the 1970s are therefore required to allow for a comprehensive comparison for pre‐ and post‐globalization intraindividual variation. In this study, the individuals born in the 1980s and 1990s (T1, T2, T6) recorded more variation in their Sr–O–C isotope ratios and lower Sr concentrations (37–88 ppm) than the individuals born in the 1940s and 1960s (D3, D13, D15; 57–103 ppm). Interestingly, one of the individuals in the younger group (T2) was a self‐reported vegetarian from the age of 10 onwards and had lower Sr concentrations than the other individuals in the same age group (T2, T6) while Sr content is reported to be higher in plants (Pate, 1994). It should be noted that not all individuals raised on supermarket diets show increased variation in ^87^Sr/^86^Sr: a total of 26 individuals born after 1972 showed little variation between ^87^Sr/^86^Sr ratios of their cusp and bulk enamel (^87^Sr/^86^SrΔ_Cusp‐Bulk_ 0.000000–0.000045, Table 4, Figure 6).

### 4.3 Increased Sr–O–C isotope variation and elevated oxygen values in carious enamel

This study indicates that caries do not seem to have a major isotopic effect on the macroscopically unaffected enamel of the same dental element, indicating that if a dental element is affected by caries it is still possible to sample the unaffected areas. Future studies should compare the results of carious dental elements to the isotopic values of an unaffected dental element from the same individual to provide more conclusive evidence, while taking into account the intraindividual variation described here.

Dental elements with caries showed twice the variation in their ^87^Sr/^86^Sr ratios (Δ_max−min_ = 0.000022–0.000449, Δ = 0.000427; avg_isovar ± 0.000162) and Sr concentrations 7 to 75 ppm (Δ = 68, avg_isovar ± 28 ppm) compared with healthy dental elements (Δ^87^Sr/^86^Sr_max−min_ = 0.000021– 0.000193, Δ = 0.000172; avg_isovar 0.000062; ΔSr ppm_max−min_ = 3–44 ppm, Δ = 41, avg_isovar ± 15 ppm). This increased variation is caused by elevated ^87^Sr/^86^Sr values in caries (*n* = 2 of 6) and increased variation in Sr concentrations and Sr isotope composition in caries (*n* = 4 of 6). Variation seen in Δδ^18^O_max‐min_ was higher on average in dental elements affected by caries than enamel from healthy individuals (1.0–1.8‰ compared with 0.4–2.0‰). The variation in carbon isotopes in dental elements affected by caries was less than in healthy individuals (Δδ^13^C_max−min_ = 0.3‰ to 1.3‰ compared with 2.1‰). The avg_isovar is similar for carbon in carious and healthy enamel, but almost twice as high in oxygen (1.2‰) due to significant elevated δ^18^O values in caries (*n* = 3 of 6). Although the increased variation in Sr isotopes and the decreased variation in C isotopes could also be explained by the small sample size, the significant differences between carious and healthy enamel in oxygen isotopes indicate that caries should not be sampled for oxygen isotopes.

Strontium concentration in caries is indistinguishable from unaffected enamel in the same dental element (see also Little & Steadman, 1966), with the exception of one caries (F6‐G2, Figure 7). The increased Sr concentrations (+ 64 ppm) and elevated ^87^Sr/^86^Sr values (Δ^87^Sr/^86^Sr = ~0.0004) in the buccal caries of F6 remain unexplained, as the other F6 caries sampled are within error of the Sr isotope compositions and concentrations of the unaffected sample locations. The results of the buccal caries of F6 cannot be explained by background information provided by the questionnaire as the individual did not report moving and it appears likely that the three caries were active at the same time.

The non‐Dutch data strongly suggest that the de‐ and re‐mineralization processes involved in the development of caries (Piesco & Simmelink, 2002; Wazen & Nanci, 2012) are not likely to incorporate Sr from the diet consumed when the caries were active. The individuals that grew up in the Dominican Republic and Somalia developed their caries in the Netherlands, yet the isotopic results (Sr, O) of both unaffected and carious enamel are representative of the Dominican Republic and Somalia, and thus distinct from the Dutch isotopic signal.

Although in most cases caries do not significantly affect the Sr–C isotope composition, the data presented here indicate that sampling caries is not recommended for modern dental elements. Oxygen isotope values are altered and the elemental incorporation process in caries remains poorly understood, as well as the time frame involved in the development of the caries.

## 5 CONCLUSION

Assessing the intradental, interdental, and population isotopic variability is a vital step in providing a framework for provenance and dietary interpretations. This work establishes that a single sample location is not representative for the total intradental enamel isotopic variation and that bulk analyses average the total variation present in the modern third molars.

This study indicates that drilled samples should be taken from the inner enamel, with no preference for a particular cusp/wall region as these locations offer comparable isotopic results. Sampling approaches should avoid carious enamel as this study indicated that caries produce inconsistent results. The unaffected enamel of carious dental elements seems to be isotopically unaltered and can be used for isotopic analyses.

Further studies are required to quantitatively evaluate the intraindividual variability in modern and archeological enamel in dental elements other than third molars, as well as the effect of caries on the isotopic composition of enamel. The intraindividual isotopic variation is expected to be controlled by a combination of the geological area in which food is grown and personal diet preferences of individuals. The resulting isotopic variation needs to be quantified for other modern or archeological populations living in regions with larger topographical and geological variation by analyzing the enamel of multiple individuals (>20) to provide a baseline to which intraindividual isotopic variation can be compared.

For Dutch modern enamel, the average isotopic variation (avg_isovar: ^87^Sr/^86^Sr ± 0.000062, Sr ppm ± 15 ppm, δ^18^O and δ^13^C ± 0.7‰) provides a more accurate estimation of the intraindividual variation than reporting the results and analytical error of a single‐locus sample. The maximal differences seen in this study should also be taken into account (^87^Sr/^86^Sr ~0.000200, 44 Sr ppm, δ^18^O, and δ^13^C ~1.4‰). Therefore, interpretations of diet and mobility should be made cautiously until isotopic variation is adequately quantified in the relevant region. For modern Dutch individuals, Sr isotope variation >0.0002 is required to argue for mobility and differences under 2‰ are negligible for δ^18^O and δ^13^C.

## Data Availability

The data that support the findings of this study are available in Tables 2‐4 as well as openly available at the 4TU.Centre for Research Data (Plomp, Verdegaal‐Warmerdam, & Davies, 2020, http://doi.org/10.4121/uuid:f6dc4f20-a6e0-4b2f-b2f8-b79a4f9061c3).
